# Management of Physical Health Conditions in Old Age Psychiatry: A Quality Improvement Project

**DOI:** 10.1192/bjo.2025.10350

**Published:** 2025-06-20

**Authors:** Tamara Chithiramohan, Umar Valera, Atish Chamunda, Jennifer Hughes

**Affiliations:** 1 Leicestershire Partnership NHS Trust, Leicester, United Kingdom; 2 University Hospitals of Leicester, Leicester, United Kingdom

## Abstract

**Aims:** Psychiatric patients on elderly psychiatric wards often have multiple physical comorbidities and there is also a clear link between mental and physical health. Trainees on old age psychiatry wards, both during the day and on call, have to manage acute and routine medical conditions. We wanted to evaluate how confident trainees find managing acute and routine medical conditions on the psychiatry wards, and whether they know where to gain advice from.

**Methods:** A survey via googleforms was designed to assess confidence in managing medical conditions in old age psychiatry patients, whether they often require advice, and if they know who to contact. We also asked which conditions they felt they required more guidance on.

**Results:** 9 responses were collected. 66.7% of trainees felt quite confident in managing day to day medical conditions, 22.2% did not feel confident at all and 11.1% were neutral. In managing medical emergencies, 11.1% felt very confident, and 22.2% felt quite confident, however 33.3% felt neutral, 11.1% felt only slightly confident and 22.2% felt not confident at all.

There was a range in responses to whether trainees tend to rely on advice from others, as opposed to managing medical conditions themselves. With 22.2% disagreeing with the statement, 33.3% neither agree nor disagree, 22.2% agreeing and 11.1% strongly agreeing.

Based upon these results, a brief guide was created with management tips of common medical problems and links to useful guidelines. A second survey was then sent out to assess usefulness.

80% of respondents felt it was very useful in both emergencies and day to day conditions, and 20% felt it was useful. 80% felt it contained information they did not already know and 100% felt it was a useful addition to the trainee handbook and it covered all medical conditions they wanted it to.

**Conclusion:** There was a range in confidence levels in managing medical conditions, however some trainees reported not feeling confident at all, or only slightly confident. Based upon these results, a brief guide to management of common medical conditions was created, for both day to day and routine medical conditions, and links to useful guidance. This was created by trainees, with advice and information provided by the ward GP. Trainees found this handbook very useful.

